# Harnessing the power of Microscale AcoustoFluidics: A perspective based on BAW cancer diagnostics

**DOI:** 10.1063/5.0180158

**Published:** 2024-02-29

**Authors:** C. L. Harshbarger

**Affiliations:** Department of Orthopedics, Balgrist University Hospital, University of Zurich, Zurich, Switzerland; Institute for Biomechanics, Swiss Federal Institute of Technology Zurich, Zurich, Switzerland; and Institute for Mechanical Systems, Swiss Federal Institute of Technology Zurich, Zurich, Switzerland

## Abstract

Cancer directly affects one in every three people, and mortality rates strongly correlate with the stage at which diagnosis occurs. Each of the multitude of methods used in cancer diagnostics has its own set of advantages and disadvantages. Two common drawbacks are a limited information value of image based diagnostic methods and high invasiveness when opting for methods that provide greater insight. Microfluidics offers a promising avenue for isolating circulating tumor cells from blood samples, offering high informational value at predetermined time intervals while being minimally invasive. Microscale AcoustoFluidics, an active method capable of manipulating objects within a fluid, has shown its potential use for the isolation and measurement of circulating tumor cells, but its full potential has yet to be harnessed. Extensive research has focused on isolating single cells, although the significance of clusters should not be overlooked and requires attention within the field. Moreover, there is room for improvement by designing smaller and automated devices to enhance user-friendliness and efficiency as illustrated by the use of bulk acoustic wave devices in cancer diagnostics. This next generation of setups and devices could minimize streaming forces and thereby enable the manipulation of smaller objects, thus aiding in the implementation of personalized oncology for the next generation of cancer treatments.

## I. INTRODUCTION

There is a plethora of cancer detection methods, such as visual inspection and tissue biopsies,^1^ cervical smears,^2^ considering family history and high risk genes,^3^ ultrasonography,^4–6^ magnetic resonance imaging,^7^ computed tomography^8^ or positron emission tomography scans,^9^ and biomarkers,^10^ to name just a few. This highlights the significant demand for diagnostic methods. Even though gold standard methods have a proven track record, they face criticism. This in seen in the case of the biomarker Prostate Specific Antigen (PSA), whereas “at best PSA screening modestly decreased prostate cancer-related mortality.”^11^ Similarly, when considering breast cancer, manual palpation,^12^ mammography,^13–15^ and ultrasound,^16^ all have demonstrated limitations with life altering consequences. Other contemporary cancer detection methods can have further drawbacks, including being highly invasive and painful, expose patients to radiation, and have limitations in detecting small tumors.^17–19^ Highly invasive solid biopsies, which at least have a high informational value compared to image based diagnostic methods, inherently possess a single biopsy bias in both time and space.^20^ There is, therefore, a pressing need for minimally invasive methods with higher information content than current imaging techniques. It would furthermore be beneficial if future methods allow for multiple samples to be taken at different time intervals to help combat cancer, a disease that will directly affect one out of three people.^21^ Even though cancer is a group of highly heterogeneous diseases,^22^ with significant inter- and even intra-patient variability,^22^ some fundamental traits have been described. Eight hallmarks of cancer are reported in the *magnum opus* work of Hanahan and Weinberg:^23^ “self-sufficiency in growth signals, insensitivity to anti-growth signals, evading apoptosis, limitless replicative potential, sustained angiogenesis, abnormal metabolic pathways, evasion of the immune system, and tissue invasion and metastasis.” Metastasis, derived from ancient Greek meaning “beyond standstill,” is the process by which cancer cells spread throughout the body, forming distant lesions, and is at the core of more than 90% of cancer related deaths.^24^ The metastatic process is nonhomogeneous between cancer types and is currently not fully understood.^25^ What is known is that cancer cells can be found in many bodily fluids, such as pleural effusions,^26^ lymphatic fluid,^27^ and cerebrospinal fluid.^28^ A bodily fluid that can contain cancer cells and is often routinely sampled, in contrast to the beforehand mentioned fluids, is blood.^29^ Cancer cells disseminate from the primary tumor and intravasate into blood vessels in order to spread throughout the body,^29^ whereas cancer cells in the cardiovascular system are termed Circulating Tumor Cells (CTCs).^30^ CTCs are exceptionally rare, approximately 1–10 CTCs per ml of blood.^31^ This is a stark contrast to the $5\times 10^{9}$ red and $10^{6}$ white blood cells per milliliter blood,^32^ as summarized in Table I.

**TABLE I.** CTCs are exceptionally rare compared to red and white blood cells in the cardiovascular system. Concentrations are given as number of cells per milliliter of blood.

	Cell concentration (#cells/ml blood)
Red blood cells^32^	5 × 10^9^
White blood cells^32^	10^6^
CTCs^31^	1–10

Even though the amount of CTCs found in blood is low, blood samples are far less invasive than solid biopsies and can be taken at predetermined intervals. This has led to the isolation of CTCs being a common research focus,^33^ as blood samples present a unique opportunity to isolate these disseminated tumor cells in order to gain patient specific diagnostic insight and to monitor treatment.^34^

Numerous methods are capable of isolating CTCs out of a blood sample, including microfluidics. Microfluidic technologies can be classified in many ways, whereas this article focuses on two core characteristics, namely, if they are label-dependent or label-free, and whether they are passive or active.^35,36^ Label-dependent technologies involve targeting a specific molecule of interest on CTCs, often using antibodies. These antibodies may be combined with secondary antibodies to add fluorescent labels,^37^ bubbles,^38^ elastomeric materials,^39^ or magnetic particles^40^ to the primary antibody and, therefore, to the target molecule. This approach relies on the presence of a suitable biomarker on the surface of the biological cell. One frequently targeted biomarker is the Epithelial Cell Adhesion Molecule (EpCAM), as cells in the circulatory system should not present EpCAM. Unfortunately, EpCAM is not exclusive to CTCs.^41^ Further biomarkers can be used to increase the specificity of cancer cell detection. Relevant biomarkers are cytokeratins (CKs), which are expressed by CTCs, and CD45, which is present on white blood cells but not on CTCs.^42^ These additional markers may not always suffice, as blood samples from healthy patients contain between 0% and 20% CK positive cells that are not CTCs.^43,44^ Furthermore, when cancer cells undergo epithelial to mesenchymal transition (EMT), thus increasing the cancer cell motility, the CTCs can lose the EpCAM marker.^45^ Last, unlike epithelial-derived carcinomas, mesenchymal-derived sarcomas, such as bone cancers, do not typically present the EpCAM marker.^46^ Thus, a label-free microfluidic method is highly desirable. Not only due to the lack of unique biomarkers but also to reduce labor-intensive tasks associated with labeling protocols that require skilled personnel and are susceptible to observer bias. Passive microfluidic devices utilize spirals,^47^ pinched flows,^48^ hydrodynamic spreading,^49^ deterministic lateral displacement,^50^ transient cellular adhesion,^51,52^ or cellular immobilization^53,54^ to isolate CTCs, to name a few. The vast amount of methods listed indicates a constant search for an optimal method of CTC isolation. Some designs introduce geometric obstacles within the microfluidic device, although one drawback of sorting by size is that the viability of biological cells can be reduced.^55,56^ Depending on the passive method, the drawbacks are low flow rates, clogging, biases introduced due to deformability differences between cells and nonspecific binding or not binding at all.^57–59^ Therefore, label-free methods with high flow rates and reduced clogging are sought after. Active methods are dependent on an external field, such as magnetic,^40,60–63^ electric,^64,65^ and optic^66,67^ fields. A large quantity of magnetic field dependent applications are based on labeling and magnetic and electric fields are, in some cases, based on the electric and magnetic properties of biological cells, limiting their applicability. Optical methods often manipulate one object at a time, leading to low throughput, which hinders their use when analyzing a large number of biological cells.

## II. MICROSCALE ACOUSTOFLUIDICS

### A. Working principal and mathematical basis

**FIG. 1.**
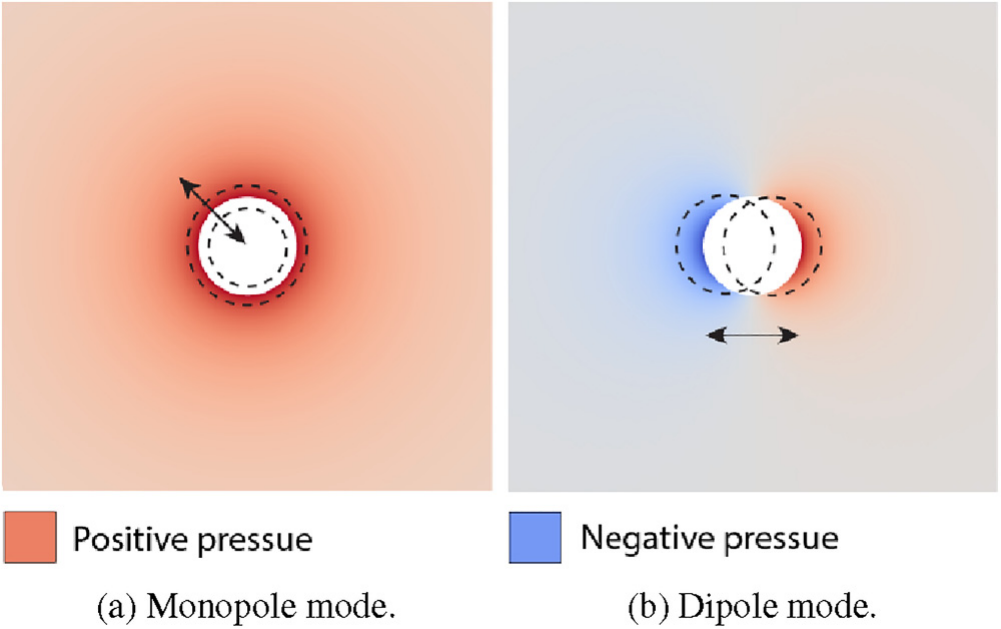
Qualitative visualization of the scattered pressure fields for the monopole and dipole mode of an object in a fluid. The white circle is the spherical object in the fluid without an external field. The dashed black circles are the change of (a) volume or (b) position of the object in the fluid caused by an incoming standing pressure wave in the horizontal direction. The arrows indicate the direction of the oscillation. Red indicates a positive pressure and blue indicates a negative pressure of the scattering fields radiating away from the object, where a darker color indicates a greater absolute pressure. The open-source OSAFT library^79^ was used to create the pressure fields, with r=1μm, f=1MHz, $c_{0}=1500\;{m\;s}^{-1}$, $\rho_{0}=1000\;{\mathrm{kg}\;m}^{-3}$, $\rho_{p}=1050\;{\mathrm{kg}\;m}^{-3}$, E=3.2GPa, ν=0.35, and $p_{A}=100\;\mathrm{kPa}$. (a) Monopole mode. (b) Dipole mode.

A further technology, which employs an external field, is Microscale AcoustoFluidics (MsAF), as acoustic fields can be exploited to manipulate the position of objects in a fluid. An extensive review^35,68^ is outside of the scope of this perspective, but to build a foundation, the core aspects of this method are introduced. This method of object manipulation in a fluid is contactless, can be label-free, and does not diminish the cell viability or proliferation.^69^ MsAF devices can be classified based on how energy is instilled into the system, which is needed to influence the position of the object in the fluid. Surface acoustic wave (SAW) devices make up one of the two large sub-classes of MsAF devices.^68^ In SAW devices, acoustic waves propagate along the surface of an elastic material, whereas these devices are versatile, compact, and inexpensive devices for contact-free object manipulation.^70^ Although SAW devices have shown much promise,^71–73^ with multiple studies on the isolation of CTCs^74–76^ and nanometer sized objects, such as exosomes,^77^ the focus of this article lies on a second large sub-class of MsAF devices, termed bulk acoustic wave (BAW) devices. In BAW devices, a voltage potential is applied to a piezoelectric transducer (PT), which produces mechanical vibrations in the solid body of the MsAF device. These whole body vibrations are translated into traveling waves in the fluid cavity. The vibrational amplitude of the PT is linearly related to the pressure amplitude in the device, whereas the acoustic energy density within the fluid cavity scales to the power of two with respect to the pressure amplitude.^78^ Precise and predicable focusing of objects in the fluid can be achieved when a standing wave is formed. The standing pressure wave, which is a result of the superposition of the traveling waves, is characterized in part by spatially fixed points of maximal and zero pressure regions. The wavelength of the standing pressure wave is determined as two times the width of the fluid cavity divided by the desired number of pressure nodes. The optimal excitation frequency for a desired amount of pressure nodes of a standing pressure wave in a BAW acoustofluidic device can, therefore, be determined via the speed of sound of the fluid in which objects are suspended divided by the wavelength with the desired amount of pressure nodes. The resulting potential in the system, termed the Gor’kov potential, can be used to predict how the objects in the fluid will interact with the pressure wave in the fluid cavity. Assuming that the object in the fluid is spherical, is a weak point scatter, is much smaller than the wavelength of the wave, and that the fluid is inviscid, the Gor’kov potential is given by

$$U=\frac{4\pi }{3}r^{3}\left[f_{1}(\tilde{\kappa })\frac{1}{2\rho_{0}c_{0}^{2}}\langle p_{1}^{2}\rangle -f_{2}(\tilde{\rho })\frac{3}{4}\rho_{0}\langle v_{1}^{2}\rangle \right],$$ (1)

where r is the radius of the object, $\rho_{0}$ is the density of the fluid, $c_{0}$ is the speed of sound of the fluid, $\left\langle p_{1}^{2}\right\rangle$ is the first order time averaged square of the incident acoustic pressure, and $\left\langle v_{1}^{2}\right\rangle$ is the first order time averaged square of the incident acoustic velocity. The scattering of the pressure waves results from the relative difference between the density and compressibility of the fluid and the object in the fluid. This is reflected in the monopole coefficient $f_{1}(\tilde{\kappa })$, which is related to the relative compressibility,

$$f_{1}(\tilde{\kappa })=1-\tilde{\kappa };\;\tilde{\kappa }=\frac{\kappa_{\;p}}{\kappa_{0}},$$ (2)

where $\kappa_{\;p}$ is the compressibility of the object in the fluid and $\kappa_{0}$ is the compressibility of the fluid and the dipole coefficient $f_{2}(\tilde{\rho })$, which is related to the relative density

$$f_{2}(\tilde{\rho })=2\frac{\tilde{\rho }-1}{2\tilde{\rho }+1};\;\tilde{\rho }=\frac{\rho_{\;p}}{\rho_{0}},$$ (3)

where $\rho_{\;p}$ is the density of the object in the fluid. Compared to the monopole mode, which is illustrated as a change in volume, a dipole radiation is, for example, emitted by a rigidly oscillating sphere, as illustrated in Fig. 1.

The corresponding force related to the interaction of the objects in the fluid with the pressure wave within the fluid cavity is termed the acoustic radiation force (ARF or $F_{ARF}$), and is commonly approximated as the negative gradient of the Gor’kov potential^80^

$$F_{ARF}=-\nabla U.$$ (4)

The objects in the fluid, according to ARF acting on them, minimize their potential by migrating to areas where their acoustic potential is minimal. $F_{ARF}$ can be simplified to one dimension (here in the y-direction),^81^

$${F(y)}_{ARF}^{1D}=4\pi r^{3}E_{ac}k_{y}\Phi (\tilde{\kappa },\tilde{\rho })\sin\left(2k_{y}y\right)e_{y},$$ (5)

where $k_{y}=\frac{\omega }{c_{0}}$ is the wavenumber in an inviscid fluid in y-direction, $E_{ac}$ is the acoustic energy density, and Φ is the acoustic contrast factor (ACF),

$$\Phi (\tilde{\kappa },\tilde{\rho })=\frac{1}{3}f_{1}(\tilde{\kappa })+\frac{1}{2}f_{2}(\tilde{\rho }).$$ (6)

**FIG. 2.**
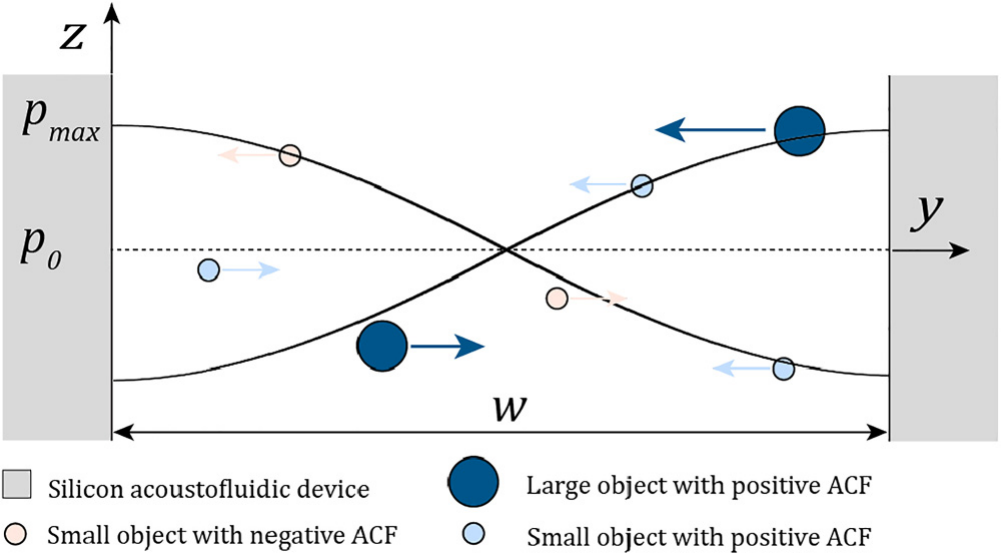
ARF of a λ-half-mode acting on small and large objects with a positive ACF and on small objects with a negative ACF. The positive ACF objects are pushed to the pressure node in the middle of the fluid cavity where the pressure is zero. The negative ACF objects are pushed away from the pressure node to the pressure antinode, where the pressure is maximal. The arrows indicate the direction of the ARF but not the precise magnitude. Reprinted with permission from C. Harshbarger, “Acoustically focusing and measuring biological cells,” Ph.D. thesis (ETH Zurich, 2023).^82^

The one-dimensional ARF can be graphically illustrated, as seen in Fig. 2.

Another relevant force in MsAF devices is Stokes’ drag force produced by the acoustic streaming (AS) velocity $v_{str}$, which acts on the object in the fluid,^83,84^

$$F_{str}=6\pi \eta rv_{str},$$ (7)

where η is the dynamic viscosity of the fluid. Stokes’ drag force is furthermore dependent on the velocity of the object in the fluid $v_{\;p}$,

$$F_{drag}=6\pi \eta r(v_{str}-v_{\;p}).$$ (8)

Hence, Stokes’ drag force contributes to the forces exerted on the object for $(v_{str}-v_{\;p})\neq 0$. As the ARF and Stokes’ drag force are two forces that can dominate MsAF devices, the particle velocity in the y-direction $v_{\;p}(y)$ can be calculated by neglecting particle inertia and by balancing the ARF with Stokes’ drag,^85^

$$v_{\;p}(y)=\frac{2\Phi }{3\eta }r^{2}k_{y}E_{ac}\sin\left(2k_{y}\left(y+\frac{w}{2}\right)\right)+v_{str}.$$ (9)

As Stokes’ drag force, no matter the origin, scales with r [Eq. (8)] and the ARF scales with $r^{3}$ [Eq. (4)], Stokes’ drag force will always contribute to the motion of the object in the fluid. However, as the object increases in size and under the assumption that the acoustic field is kept constant, the contribution of Stokes’ drag force stemming from the acoustic streaming velocity is diminished in relationship to the contribution of the ARF. Therefore, there is a radius, termed the critical radius, above which the ARF cannot overcome Stokes’ drag force stemming from the object motion $v_{\;p}$, but can overcome Stokes’ drag force resulting from the acoustic streaming velocity $v_{str}$.^86^

### B. Applications

**FIG. 3.**
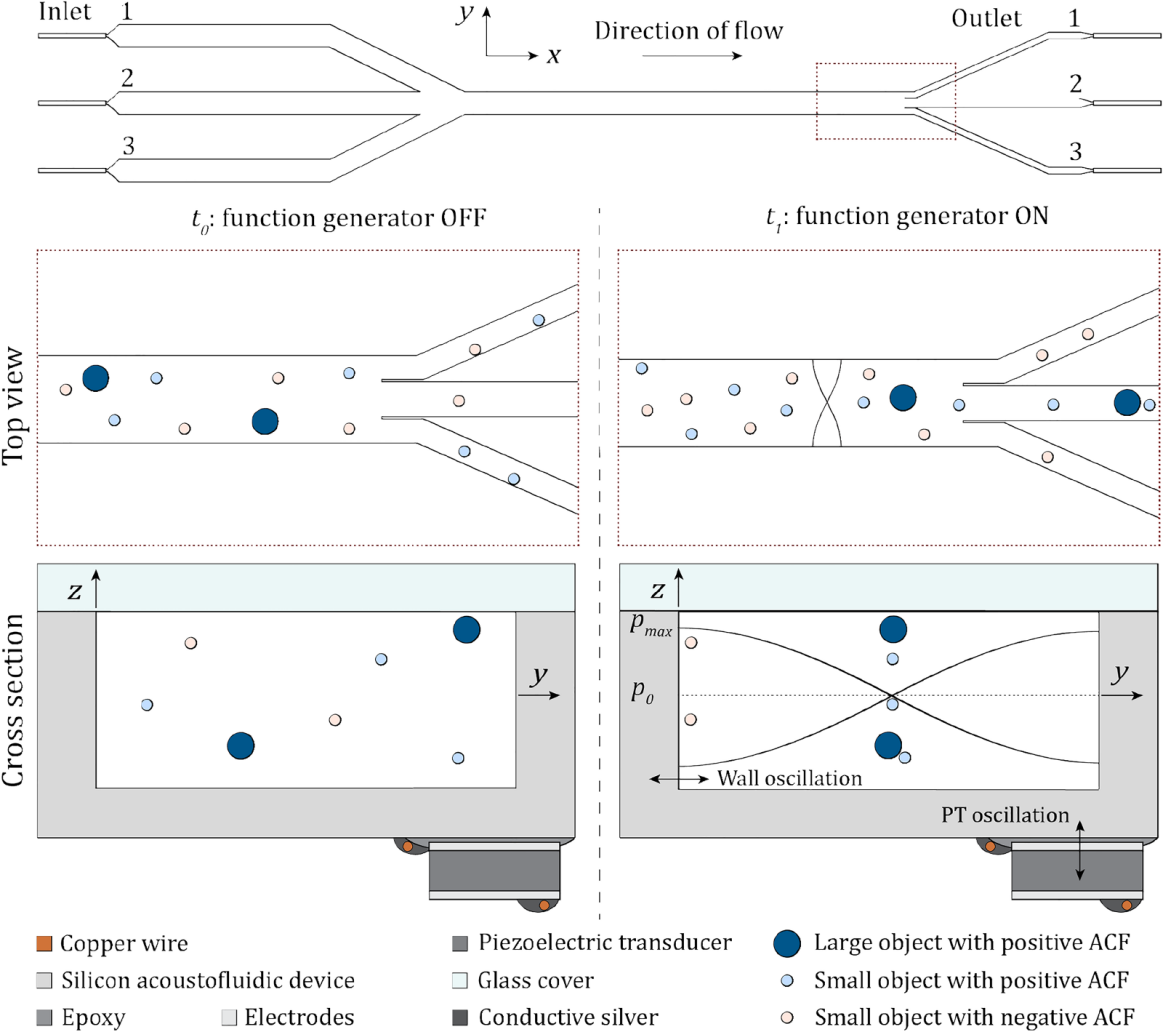
Schematic of a typical BAW acoustofluidic device with multiple in- and outlets. There are three inlets (left hand side), which all can be filled with either a sample solution or buffer fluid. The fluid flow is in the positive x-direction, and the fluid and objects in the fluid can exit via one of the three outlets (right hand side). The cross section shows a generic BAW device with a top cover made out of glass and a silicon body in which a fluid cavity has been etched. The PT is glued to the silicon base material via a conductive epoxy. The copper wires are electrically and mechanically connected via conductive silver. The solution in the fluid cavity contains small (light blue) and large (navy blue) objects with positive and small (salmon) objects with a negative acoustic contrast factor (ACF). At $t_{0}$, no voltage is being applied to the PT, and, therefore, the PT is not oscillating; thus, the objects are randomly distributed in the fluid and leave through any outlet. At $t_{1}$, the function generator is turned on and the PT starts to oscillate. This causes the wall to oscillate and, given the correct excitation frequency, a standing wave is formed. This forces the objects to the pressure nodes, if they have a positive ACF, or to the antinodes, if they have a negative ACF, whereas the larger objects experience a larger force and thus move faster. If a λ/2-mode is excited, the positive ACF objects exit the device through outlet 2 and the negative ACF objects exit through outlets 1 and 3. Important to note is that at $t_{1}$ the top view only shows one standing pressure wave location, although the standing wave is present within the whole length of the fluid cavity. The schematic is not drawn to scale. Reprinted with permission from C. Harshbarger, “Acoustically focusing and measuring biological cells,” Ph.D. thesis (ETH Zurich, 2023).^82^

The general working principle of a BAW device is illustrated in Fig. 3. Biomedical applications utilizing BAW devices include the formation of cell spheroids,^87^ cell media exchange,^88^ separation of living and dead cells,^89^ and cell patterning for tissue bioengineering.^90,91^ Another application of BAW devices is the isolation of single cell (sub-)populations, for which the foundation was laid in the early 2000s.^92–97^ From these concept studies, strides toward isolating cancer cells from liquid biopsies have been made, such as using elastomers with a negative ACF that are attached to biological cells,^39^ which, however, is not label-free. Harnessing the power MsAF, however, includes refraining from label-dependent solutions. The label-free isolation of CTCs from CTC spiked red blood cell-lysed human blood has been demonstrated by a large group of publications,^98–100^ whereas the isolated CTCs can be used for downstream analysis. These studies were, however, limited to research environments. A, not comprehensive, list of limitations hindering a widespread use outside of the research environment is unknown dynamic material properties of the biological cells to be isolated, CTC clusters not being considered in the study design, a lack of small and automated setups, and the size of objects that can be manipulated. The silver lining is that all of these current limitations can be overcome.

## III. TOWARD FULLY HARNESSING THE POWER OF MICROSCALE ACOUSTOFLUIDICS

Tackling the mentioned limitations could propel MsAF out of the research environment, as illustrated by this perspective article highlighting the use of BAW devices for cancer detection, where ideas are presented in the field of measuring mechanical properties, single CTCs vs CTC clusters, setup modifications and automation, and decreasing the critical size of the object in the fluid.

### A. Mechanical properties

One material property of cancer cells that has gained relevance is the stiffness of CTCs. Cancer cells are known to decrease their stiffness when they become more malignant.^101^ The mechanical properties of biological cells are non-trivial to measure and, depending on which gold standard method is used, the values can span three orders of magnitude.^102^ MsAF cannot measure the stiffness of the CTCs directly, but the ARF is related via Eq. (3) to the compressibility of the object in the fluid. This on chip measurement has been exploited^39,103–108^ and the discussion on how the malignancy is related to the compressibility is ongoing.^108^ Continuing this line of investigation holds significance for two reasons, as isolating CTCs from a liquid biopsy is only the first step. First, understanding the material properties of CTCs is crucial for designing effective MsAF devices. Second, there can be orders of magnitude between static and dynamic material properties,^109^ whereas the dynamic material properties are currently often times not available. Overcoming this lack of material properties might prove vital for upcoming potential cancer treatment methods, such as oncotripsy,^110^ ultrasound neuromodulation,^111,112^ and sonogenetics.^113^ Even in the cases where material properties are measured, there is no standard within the community of which values to report and what reference values are taken. To overcome this, a consensus in the community of how the material properties are measured and which values are reported must be found.

### B. CTC clusters

In addition to altered mechanical properties, a decrease in the size of the cell has been linked to an increase in their malignancy for some cancer cells.^114^ Equation (6) indicates a cubic relationship between the ARF and the radius of a cell, therefore yielding smaller, and potentially more malignant cells, harder to isolate, especially as white blood cells are more numerous by multiple orders of magnitude and have very similar ACFs and size range when compared to CTCs. Not only are CTCs incredibly rare, 1–10 per milliliter of blood,^32^ new research shows that CTC clusters might be the driving factor of metastasis.^115,116^ MsAF could prove particularly useful in isolating these CTC clusters, as the ARF scales with the volume of the object, thus allowing for a rapid isolation of such clusters due to their relative size compared to other cells in the circulatory system.

### C. Setup modifications and automation

Current MsAF setups often make use of optical setups. These optical setups are prevalent as a live video feed is easy to calibrate and provides direct feedback. The trade-off is that these optical setups can be expensive, highly customized for one task, and have a large space requirement. A reduction of the cost, complexity, and space required could be reached by shifting away from classical optical setups. The optical setup can be replaced by a light sheath and a detector. This would necessitate a more common use of see-through devices but could increase the reliability of the analysis of the MsAF performance due to fewer artifacts and latency compared to a video feed. Furthermore, handheld acoustic nebulizers^117,118^ could act as inspiration for miniaturization, and the electrical circuit needed to drive the PT on the BAW device could integrated onto a chip.^119^ Implementing these proposed changes could make BAW devices more user friendly, efficient, and reduce the dependency on highly trained operators which combined could lead to bedside applications. This would, however, necessitate that the optimal excitation frequency to establish the standing waves would be maintained. The theoretical resonance frequency is hard to accurately predict, even when increasing the production process and numerical optimization of such devices.^120–124^ Instead of trying to predict the performance *a priori*, a feedback control loop (FCL) can be implemented. This idea dates back to 1996,^125^ with only few implementations reported.^126–130^ A FCL can be used to find and maintain the optimal frequency for object focusing, demonstrating in flow manipulation of sub-micrometer objects.^131^ A FCL could additionally be used to even further increase the sample throughput of already optimized devices^132^ or enable multiplexing in MsAF devices.^133^

### D. Decreasing the critical size of the object in the fluid

Decreasing the object size is a delicate task, as the vibration of the PT induces traveling waves and acoustic streaming (AS). AS becomes important for smaller objects, as the AS will induce a movement of the object and, thus, result in a contribution to Stokes’ drag force as detailed in Eq. (8), hindering the precise positional manipulation of the object in the fluid. Optimizing a FCL, factoring in the known behavior of MsAF devices, for instance, the buildup time of the acoustic radiation force,^134^ making use of MsAF devices with built-in streaming suppression,^135–137^ and studies on the multibody dynamics in acoustic devices^138,139^ could further decrease the size of objects that can be manipulated in MsAF devices. Decreasing the size is highly relevant as MsAF could potentially be used for applications in the isolation of circulating tumor (ct)DNA^140^ or circulating microRNA^141^ and is an active field of research^142^ within the MsAF community. The detection of ctDNA is useful as it can be used to detect cancer before it is clinically relevant.^143^ In addition, diagnostic approaches are heavily reliant on a histological analysis of a biopsy. However, increasing capabilities in genetic analysis allows for a more patient-specific approach of cancer treatment under the umbrella term of personalized oncology.^144^ This new approach is, however, only viable if the necessary data, gained through CTCs, CTC clusters, circulating microRNA, or ctDNA, to name but a few possibilities, can be collected in a non-biased way.

### E. Closing remark

The above sections indicate that there is much to be done in the realm of MsAF in order to fully harness its potential. Some changes are hardware based, such as the electrical and mechanical components addressed when considering the setup. Other limitations will require a shift toward automated devices and setups, which should result in an increased reliability and repeatability. Furthermore, the importance of the mechanical properties of CTCs is hard to understate. Here, a consensus needs to be found within the community of how to measure, what to measure, and what references are suitable. In addition, there needs to be a shift away from the mono-focus on individual CTCs to include CTC clusters in order to match the change of paradigm in the cancer biology community. Finally, reducing the critical size is one of the greatest challenges that needs to be addressed, but when successful can help open up new avenues in cancer diagnostics.

As cancer is mostly caused by genomic instability,^145^ life will always be faced with cancer, its spreading, and ultimately cancer related death. MsAF will not cure cancer, but pushing this technology by questioning if contemporary research focused on single CTCs and not on CTC clusters is the correct approach and by consciously improving all aspects of the devices as detailed, MsAF could help improve the early diagnosis and treatment monitoring in an effort to reduce the impact of one of the largest health concerns of the century.

## Data Availability

Data sharing is not applicable to this article as no new data were created or analyzed in this study.
